# Role of Microglia and Astrocytes in Alzheimer’s Disease: From Neuroinflammation to Ca^2+^ Homeostasis Dysregulation

**DOI:** 10.3390/cells11172728

**Published:** 2022-09-01

**Authors:** Giulia Di Benedetto, Chiara Burgaletto, Carlo Maria Bellanca, Antonio Munafò, Renato Bernardini, Giuseppina Cantarella

**Affiliations:** Department of Biomedical and Biotechnological Sciences, Section of Pharmacology, University of Catania, 95123 Catania, Italy

**Keywords:** astrocyte, calcium homeostasis, disease, microglia, neuroinflammation

## Abstract

Alzheimer’s disease (AD) is the most common form of dementia worldwide, with a complex, poorly understood pathogenesis. Cerebral atrophy, amyloid-β (Aβ) plaques, and neurofibrillary tangles represent the main pathological hallmarks of the AD brain. Recently, neuroinflammation has been recognized as a prominent feature of the AD brain and substantial evidence suggests that the inflammatory response modulates disease progression. Additionally, dysregulation of calcium (Ca^2+^) homeostasis represents another early factor involved in the AD pathogenesis, as intracellular Ca^2+^ concentration is essential to ensure proper cellular and neuronal functions. Although growing evidence supports the involvement of Ca^2+^ in the mechanisms of neurodegeneration-related inflammatory processes, scant data are available on its contribution in microglia and astrocytes functioning, both in health and throughout the AD continuum. Nevertheless, AD-related aberrant Ca^2+^ signalling in astrocytes and microglia is crucially involved in the mechanisms underpinning neuroinflammatory processes that, in turn, impact neuronal Ca^2+^ homeostasis and brain function. In this light, we attempted to provide an overview of the current understanding of the interactions between the glia cells-mediated inflammatory responses and the molecular mechanisms involved in Ca^2+^ homeostasis dysregulation in AD.

## 1. Introduction

Alzheimer’s disease (AD) is an aggressive neurodegenerative disorder and the leading cause of dementia in the elderly population worldwide, characterized by impairment of cognitive functions including memory, decision-making, orientation to physical surroundings and language [1,2]. The acknowledged neuropathological hallmarks of AD include extracellular senile plaques, composed of amyloid-beta (Aβ) peptides, and intracellular neurofibrillary tangles (NFTs) generated by the hyper-phosphorylated form of the tau protein, as well as selective neuronal and synaptic loss in specific brain regions.

To date, despite the extensive research efforts, there are no disease-modifying therapeutic options and the currently available Food and Drug Administration (FDA) approved pharmacotherapy for AD provide only a temporary symptomatic relief, without affecting pathophysiological disease progression. Due to these results, researchers are attempting multifaceted and integrated approaches, ranging from drug repurposing to stem cell technologies [3,4]. While compelling evidence suggests that AD has a multifactorial aetiology [5], a robust glial-mediated inflammatory response manifests as an early feature of AD pathophysiological mechanisms and plays a pivotal etiopathogenic role [6,7], due to the ability to exacerbate Aβ and Tau pathologies [8,9]. The inflammatory response in AD acts as a “double-edged sword”. In fact, while on the one hand, during the initial phases of AD, neuroinflammation represents a self-defence mechanism that protects the brain, by promoting tissue repair and quick clearance of noxious stimuli; on the other, as the disease progresses, sustained inflammatory response brings about detrimental effects, fuelling neurodegeneration [10,11]. Despite the neuroinflammatory events being not exclusively limited to the brain, as peripheral immune dysfunctions occur early in the disease, perhaps even prior to relevant pathogenic cerebral changes [12,13], glial cells, especially microglia and astrocytes, make up the brain’s innate immune system, engage in a fine-tuned and tight crosstalk, and represent the major players in the ongoing neuroinflammatory response in AD [14]. Remarkably, a high number of reactive astrocytes and activated microglia are found near the senile plaques of individuals with AD, suggesting their crucial involvement in AD pathogenesis [15,16,17]. Nevertheless, it is also known that an aberrant calcium (Ca^2+^) signalling represents another hallmark of AD and is reported not only in neuronal cells, but also in astrocytes and microglia [18,19]. Glial cells profoundly contribute to the inflammatory response that, in turn, impacts on neuronal Ca^2+^ homeostasis and brain function [14], leading to age-related cognitive decline. Indeed, intracellular Ca^2+^ is a second messenger that regulates many facets of neuronal physiology, including neurotransmitter release, growth, differentiation, and synaptic plasticity, as well as pathophysiological processes, such apoptosis, necrosis, and degeneration [20].

**Figure 1:**
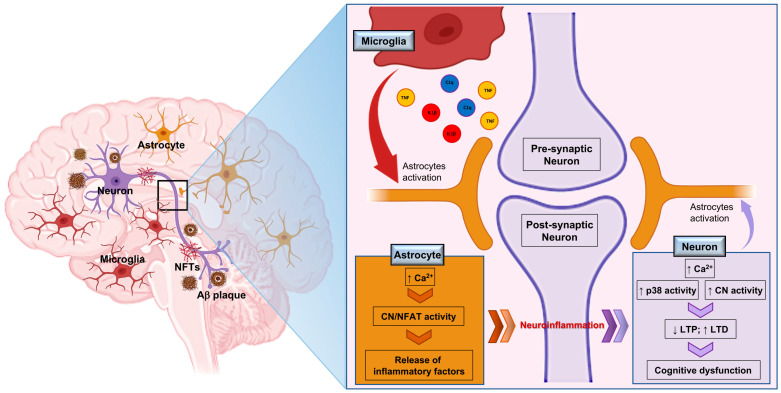
Crosstalk between Ca^2+^ dysregulation and neuroinflammation in Alzheimer’s disease. Among many other possible causes, the accumulation of amyloid-β (Aβ) plaques and neurofibrillary tangles (NFTs) in the AD brain, besides inducing microglia activation, bring about Ca^2+^ dysregulation in astrocytes. Ca^2+^ dysregulation increase calcineurin (CN) activity in astrocytes with AD, where CN assumes control over gene regulation through the activation of nuclear factor of activated T cells (NFAT) transcription factors, leading to the production and release of several inflammatory mediators, such as cytokines. In turn, inflammatory factors induce Ca^2+^ dysregulation and activation of the MAP kinase signalling pathways (p38) in neurons, leading to increased CN activity. Hyperactivation of CN in neurons determines transcriptional repression of several genes, especially those related to synapse function and integrity, causing synaptic plasticity deficits, neurite degeneration, and cognitive impairment. These parameters further participate in the activation of nearby astrocytes leading to the dysregulation of astrocytic Ca^2+^ and hyperactivity of astrocytic CN that, through the regulation of the transcriptional induction of genes, sustain glial activation and neuroinflammation, negatively affecting neurons and exacerbating neuronal Ca^2+^ dysregulation. Perpetuation of this vicious cycle drives the progression of AD pathology.

Ca^2+^ dysregulation and neuroinflammation exhibit an extensive and tight crosstalk, leading to distinct outcomes in different neural cell types [21]. Ca^2+^ dysregulation in glial cells, with special regard to astrocytes, enhances the activity of Ca^2+^ dependent protein phosphatase calcineurin (CaN), which shows elevated activity in animal models of aging and disease, resulting in increased production and release of several inflammatory mediators, such as cytokines (i.e., interleukin (IL)-1β and tumour necrosis factor-α (TNF-α) [21]. In turn, pro-inflammatory cytokines, produced and released by astrocytes and microglia, sustain activation of the MAP kinase signalling pathways (p38), and the expression/activity of L-type voltage sensitive Ca^2+^ channels in neurons, causing Ca^2+^ dysregulation, hyperactive calcineurin activity, and synaptic plasticity deficits [21,22] (Figure 1).

In such scenario, we focused on the crucial involvement of glia cell-mediated inflammatory responses and calcium homeostasis dysregulation in AD, with the aim to identify new and promising molecular targets for the development of innovative therapies for this disease.

## 2. Neuroinflammatory Scenario in Alzheimer’s Disease: The Role of Microglia and Astrocytes

### 2.1. Microglia and Astrocytes: The Main Actors of the Neuroinflammatory Response

Over the years, several hypotheses have been proposed to unveil the complex pathological mechanisms underlying AD-related neurodegeneration [23]. However, the ultimate aetiology of AD remains obscure.

The discovery of elevated levels of inflammatory markers associated with cognitive decline in AD patients and the identification of AD risk genes associated with innate immune functions suggest that neuroinflammation holds a crucial role in AD pathogenesis [24]. Neuroinflammation not only occurs in an early phase of the disease, but may also drive its progression [25].

The main immune cell components of the central nervous system (CNS) microglia and astrocytes, represent the cellular master regulators of inflammatory responses in the brain [10], and as resident innate immune cells, are strongly implicated in aberrant molecular pathways that underlie AD pathogenetic alterations [26]. Traditionally, both types of cells can assume multiple reactive phenotypes, neurotoxic and neuroprotective, which are related to the stage of the disease and to the regional location [10].

Such complexity in their distribution could be one of the reasons why clinical trials of anti-inflammatory therapies, to date, have failed to improve clinical outcomes of AD patients [27]. In this light, a better understanding of the roles of microglia and astrocytes in AD is essential for developing effective therapies.

### 2.2. Microglia: The Sentinels of the Brain

Microglia cells, the brain tissue macrophages, represent slightly less than 10% of CNS cells. Nevertheless, these innate immune cells appear as the principal responders to both immunological and neuronal stimuli. In fact, microglial cells are involved in homeostasis and immune surveillance of the brain: they act as “sentinels” detecting changes in the surrounding environment by means of a cluster of genes termed “sensome” [28], migrate to injured sites and protect against noxious stimuli, producing cytokines and chemokines to recruit additional cells and remove pathological agents [10,29,30]. Microglia are also active players in complex neurodevelopmental programs such as neurogenesis and synaptic pruning [31]. In the adult brain, microglia interact with neurons and macroglia cells to provide trophic support, synaptic modulation, and neuronal reorganization [32,33]. Loss of homeostasis or tissue changes occurring in AD, induce various dynamic microglial processes, including changes in cell morphology (ramified or quiescent/resting, activated or ameboid), surface phenotype and proliferative responses [34].

Microglia have a complex role in the trajectory of AD owing to their diverse phe-notypes and variety of activation pathways [24]. In fact, the role of microglia in neurodegeneration related to AD evolves with the progression of the disease, revealing the complexity of the factors regulating the balance between its beneficial and detrimental effects. Microglia express a family of innate immune cell receptors, the pattern recognition receptors (PRRs) (i.e., receptors for advanced glycation end (RAGE), Toll-like receptors (TLRs), and scavenger receptors). Different Aβ species, including neurotoxic oligomeric Aβ, can activate microglia by binding to PRRs, suggesting that accumulating Aβ, is responsible for the neuroinflammation observed in AD [35,36]. In the early disease stages, the activation of microglia has been associated with neuroprotective effects by removing the harmful stimuli represented by hyper-production of Aβ, clearing cell debris, phagocytosing dead cells, and releasing neurotrophic factors [37]. However, the persistence of the damaging stimuli through the disease continuum, subjects these cells to a state of chronic activation and inflammatory cytokines release, that drives neurotoxicity and neurodegeneration. Danger-associated molecular patterns (DAMPs), that arise from these processes, further activate microglia initiating a self-perpetuating proinflammatory cascade that leads to Aβ clearance ability [29,38]. In addition, microglia seem to be intimately involved also in the propagation of tau pathology; however, it is still unclear whether microglia contribute to tau pathology by failing to engulf it or by releasing factors that exacerbate tau pathology [34]. Thus, identifying the role of disease-specific microglial phenotypes during AD progression may be of interest in designing immunotherapy that boost or temper inflammation depending on the disease stage.

### 2.3. Astrocyte: A Crucial Regulator of Brain Homeostasis

Astrocytes are the most represented glial cells in the CNS [39]. They participate in neurovascular coupling and in the integrity maintenance of the blood–brain barrier (BBB), regulate cerebral blood flow, and mediate synaptic formation and transmission [40]. Astrocytes constitute the parenchymal part of the BBB and regulate the establishment of endothelial cell-to-cell junctions that preserve the structural and functional integrity of the BBB through secretion of soluble factors, such as growth factors (i.e., glial cell-line neurotrophic factor (GDNF), vascular endothelial growth factor (VEGF), basic fibroblast growth factor (bFGF), angiopoetin-1 (ANG-1), morphogens (Sonic hedgehog (Shh) and Wnt) and extracellular vesicles [41,42].

The astrocyte–endotheliocyte communication is crucial in various neurological diseases associated with BBB impairments. In physiological circumstances, perivascular astrocytic end-feet are not tightly sealed [41]. On the contrary, in neuroinflammatory conditions related to AD, and characterized by BBB breakdown and leukocyte infiltration into the CNS, end-feet of reactive astrocytes mount a parenchymal line of defence by inducing tight junctions formation in response to inflammatory cues, thereby tightening the border to limit brain infiltration of peripheral immunocyte [43]. In this scenario, astrocytes activity may exacerbate inflammatory reactions and tissue damage, or promote immunosuppression and tissue repair depending on timing and disease context [44].

Astrocytes also exert neuroprotective function in AD, blunting the plaque build-up by Aβ clearance [45]. Conversely, astrocytes are induced by noxious stimuli, Aβ or activated microglia. Activated microglia release specific astrocyte-activating signals, such as interlukin-1 alpha (IL-1α), complement component 1q (C1q) and TNF-α, which, in turn, activate β-secretase and γ-secretase activity, cleaving APP, and stimulating β-amyloid formation by astrocytes, thereby supplementing neuronal β-amyloid production [26,46]. Therefore, inflammatory stimulation of astrocytes can induce increased expression of β-site APP-cleaving enzyme (BACE1), APP, and β-secretase, implying a feed-forward mechanism of astrocytic Aβ production [47,48].

### 2.4. Microglia and Astrocytes Activation Profile in Alzheimer’s Disease Is Heterogeneous

Although traditionally classified as either proinflammatory (neurotoxic), or immunoregulatory (neuroprotective), the phenotypic distribution of microglia and astrocytes can change based on the progression of the disease [10]. Microglia and astrocytes become activated around the senile plaques in AD brain, and the resulting morphological changes are typical of an Aβ-induced neuroinflammatory response that, in turn, induce neurodegeneration [17,49,50]. Consistently with this evidence, glial activation and subsequent inflammatory events are not only a simple response to Aβ deposition, but rather represent a key contributor to AD pathogenesis [51]. Thus, a deep understanding of the precise role of glial cells during disease progression appears essential for developing novel and effective therapies.

### 2.5. The Neuroinflammatory Cycle: Microglia and Astrocytes Crosstalk in Alzheimer’s Disease and Potential Therapeutic Approaches

Neuroinflammation is the result of a complex of responses generated by a network of interactions between cells, such as neurons, microglia, astrocytes, and brain infiltrating leukocytes [26]. Particularly, microglia and astrocyte reactions encompass pro- and anti-inflammatory responses which attempt to defend neurons from damaging stimuli (i.e., pathogens and misfolded proteins), simultaneously promoting neuronal repair. Moreover, glial activity represents a self-limiting defence reaction aimed to contain and eradicate threats from the host organism [52]. Despite this, aging and neurodegenerative disorders such as AD determine functional alterations of microglia and astrocytes, subjecting these cells to a state of chronic activation. Persistent inflammation evolves when the initial acute inflammatory response is not adequately resolutive, leading to the accumulation of TNF, IFNs, and IL-6, as well as immunocytes, apoptotic cells, and debris at the site of insult/injury, thus compromising functional homeostasis [7].

Moreover, astrocytes and microglia release several signalling molecules (i.e., growth factors, neurotransmitters, cytokines, chemokines) establishing a bidirectional crosstalk for a tight reciprocal modulation to support neuronal survival and function during CNS insult or injury [33,53].

Specifically, upon injury or disease, damaged neurons release self-antigens or modified proteins that activate resting microglia. The latter, as the major immunological effector of the innate immune system in the brain and first line of defence, migrate to sites of damage to engulf dead cells and debris [54]. When inflammatory/pathogenetic stimuli persist, microglia remain trapped in a vicious cycle, characterized by a state of “non-functional” chronic activation. Activated microglia also release astrocyte-activating signals, including IL-1α, TNF-α, TNF-related apoptosis-inducing ligand (TRAIL) and C1q, which induce neuroinflammatory astrocytes that, in turn, amplify the neurodegenerative cycle [26,55].

The above reported bidirectional interactions set the stage for an endless cycle accelerating disease progression which could represent a relevant target to open new avenues into devising the long-due, disease-modifying therapies for AD.

Current evidence generally supports a simplified model of neuroinflammation in which the chronic inflammatory background provides an initial mild stimulus that results in glial priming, tied to a possible protective wave for such activated glia in the early stages of AD, along with the emergence of Aβ burden [24]. Nonetheless, the ineffective clearance of Aβ, combined with tau aggregation, impair these defence properties, and elicit an ongoing harmful glial activation processes over the course of AD [56].

Nevertheless, the huge plethora of different functions exerted by glial cells, combined with the available evidence suggesting the presence of distinct patterns of activation and the lack of a proper knowledge regarding the molecular regulations underlying cellular transitions, delay the development and design of pharmacological approaches targeting these specific glial subtypes [57,58]. This aim could be pursued through different ways, including the specific suppression of glial pro-inflammatory properties and the modulation of phenotypic changes in order to counteract microglial priming and to maintain protective and phagocytic properties for prolonged periods [59].

Several preclinical studies and phase I/II clinical trials assessing the targeting of Triggering Receptor Expressed on Myeloid cells 2 (TREM2) [60], NOD-like receptor protein 3 (NLRP3) [61], TLRs [62,63], or CD33 [64] have shown encouraging results. However, though promising, these potential therapeutic approaches are still in their early phases and further efforts are needed to develop a deeper understanding regarding the tight relationship between the mechanistic and regulatory functions of different astrocytic and microglial subtypes and their precise contributions in different stages of AD progression.

## 3. The Relevance of Ca^2+^ Homeostasis on Microglia and Astrocytes

### 3.1. Intracellular Ca^2+^ Concentration ([Ca^2+^]_i_): A Key Read-Out of Glial Activity

Evidence demonstrates that glial cells undergo complex changes in intracellular Ca^2+^ concentration ([Ca^2+^]_i_), which are considered a key read-out of glial activity and reactivity [65,66]. Glial [Ca^2+^]_i_ is four to five orders of magnitude less than that in the narrow system of clefts that constitutes the extracellular environment of the CNS [67,68]. Indeed, a steep electrochemical gradient supports the entry of Ca^2+^, causing a transient increase in cell membrane permeability to Ca^2+^ [69]. Glial cells exhibit a dynamical regulation of intra- and intercellular content of Ca^2+^, both in their steady state and in response to specific stimuli [70]. In fact, Ca^2+^ stores within the cell may release Ca^2+^ in response to specific intracellular messengers, increasing [Ca^2+^]_i_ [71]. Such transient rises of [Ca^2+^]_i_, due to the presence of Ca^2+^-permeable membrane ion channels, regulate several intracellular events, such as metabolic processes, gene expression, and ion transport systems [72]. Moreover, changes in [Ca^2+^]_i_ can be considered as a second messenger system which coordinate the changes occurring in the extracellular environment with intracellular processes [73].

### 3.2. The Versatility of Ca^2+^ as an Essential Driver of Biological Processes in CNS

Glial cells express a variety of ion channels, and neurotransmitter transporters and receptors, that enable them to detect and respond to neuronal and synaptic activity [74,75]. In physiological conditions, receptor-mediated stimuli or electrical pulses in neurons generate different Ca^2+^ signals, with distinct spatial dimensions, amplitude, oscillations, temporal extension, and subcellular localization [76,77]. Spatial variability ranges from nanodomains in cellular organelles, up to gradients which cover the whole cell body. Changes in glial [Ca^2+^]i have been measured under various conditions, as glial cells are responding to chemical, mechanical and electrical stimuli [78]. Fluctuations in [Ca^2+^]i are not only a passive response, but seem also correlated with the reaction of glial cells to environmental changes, which, in turn, lead to a modification in glial function; therefore, these are not passive responses and can be rather considered as a form of glial excitability Ca^2+^-mediated [76,77]. The versatility of Ca^2+^ as an intracellular messenger is derived from different cytosolic Ca^2+^ concentrations, generating by regulated openings of Ca^2+^-permeable channels expressed both in plasma membrane and in different organelles [71]. *Indeed,* it is known that the activation of microglial cells increases Ca^2+^ permeability, which leads to Ca^2+^ influx into the cell [79,80]. Such an increase in [Ca^2+^]_i_ acts as a second messenger that initiates signalling cascades, driving to essential biologic processes, such as cell proliferation, differentiation, migration, as well as activation of intracellular enzymatic pathways involved in transcriptional regulation of various genes [81].

Several intracellular organelles contain Ca^2+^ concentrations higher than the surrounding cytoplasm, acting as Ca^2+^ stores, but, in most mammalian cells, the major intracellular Ca^2+^ pool is the endoplasmic reticulum (ER) [71]. In microglial cells, the release of Ca^2+^ from the intracellular stores is controlled both by Ca^2+^ itself and by messengers, such as inositol-1,4,5-triphosphate (IP3) [81]. The stimulation of different receptors leads to the activation of phospholipase C (PLC), which hydrolyze phosphoinositide-4,5-bisphosphate (PIP2) to IP3 and 1,2-diacyl-glycerol (DAG). IP3 in turn induces the release of Ca^2+^ from intracellular stores, and DAG is associated with the entry of Ca^2+^ from the extracellular compartment. The IP3 interacts with its receptor (IP3R) on the ER walls, leading to the release of stored Ca^2+^ into the cytosol [82]. Another receptor for Ca^2+^ release located in the membrane of the ER is the ryanodine receptor (RyR) [83]. RyRs are regulated by multiple factors, including Ca^2+^ itself and the intracellular messenger cyclic adenosine diphosphate ribose (cADPR) [84]. In vitro studies on cultured human microglia from both foetal and adult brain specimens revealed the expression of mRNA for RyR1 and RyR2, whereas RyR3 mRNA was detected only in foetal microglia [85].

Besides IP3, the two other intracellular second messengers, cADPR and nicotinic acid adenine dinucleotide phosphate (NAADP), produced by the ADP-ribosyl cyclase CD38, are also involved in the mobilization of Ca^2+^ from intracellular stores [86]. RyRs are cADPR targets, whereas the receptor for NAADP is still unknown. NAADP could serve as a regulator for RyRs, two-pore channels, and transient receptor potential channel, subtype mucolipin (TRP-ML) channels [87,88]. The functional roles of cADPR have been well studied in microglia. In fact, several studies report that cADPR promotes microglial activation and secretion of proinflammatory cytokines [86,89,90]. On the other hand, little is known of NAADP and further studies are needed to clarify the role of NAADP in the regulation of Ca^2+^ homeostasis in microglial cells [88].

### 3.3. Mechanisms Mediating Ca^2+^ Exit from the Microglial Cytosol: The Role of Pumps and Exchangers

There is ample evidence showing that Ca^2+^ entering microglial cells through various routes, has to be finely tuned, as the accumulation of intracellular Ca^2+^ would lead to cell dysfunctions [79,91]. Mechanisms mediating Ca^2+^ exit from the cytosol involve various pumps and exchangers on the plasma membrane [92]. They include Na+/Ca^2+^ exchangers (NCX), Na+/Ca^2+^-K+ pumps (NCKX), and plasma-membrane Ca^2+^ ATPase pumps (PMCAs) [93,94]. In contrast to the abundancy of reports of NCX functions in microglial cells, little information is available on the properties of microglial Ca^2+^ pumps. It is known that NCX can operate either in the forward mode, coupling the uphill extrusion of Ca^2+^ (three ions) to the influx of Na^+^ (one ion), or in the reverse mode, coupling the extrusion of Na^+^ to the influx of Ca^2+^ ions [95,96]. Currently, three NCX genes (NCX1, NCX2, and NCX3), have been identified; in addition, it was reported that NCX1 is the most represented subtype in microglia [97].

### 3.4. Astrocyte Excitability Depends upon Variations of Cytosolic Ca^2+^ Concentration

Among glial cells, astrocytes were first characterized as non-excitable cells of the central nervous system, although expressing voltage-gated channels [98]. Astrocyte excitability results, in fact, from variations of cytosolic Ca^2+^ concentration [99]. At the cellular level, those Ca^2+^ signals occur in astrocytes in response to synaptic activity and may cause release of molecules such as glutamate, ATP, TNF-α, or D-serine, which modulate synaptic transmission [100,101,102,103], as well as vasoconstriction/vasodilatation [104,105,106,107]. The tight association of astrocytes to pre- and post- synaptic elements, both structurally and functionally, is referred to as tripartite synapse [108,109,110]. In addition, astrocytic Ca^2+^ signals can modulate neuronal synchronization and firing pattern [111,112,113] and have been observed in vivo, in response to external stimuli [114,115]. Astrocytes possess a complex Ca^2+^ signalling machinery that relies on several Ca^2+^ mobilizing pathways associated with ER Ca^2+^ release and plasmalemmal Ca^2+^ entry through ion channels and the reversed NCX [116].

The remarkable heterogeneity of astroglial Ca^2+^ signalling is strictly associated to the extensive adaptive potential of astrocytes, which may tailor Ca^2+^ signalling toolkits to meet a multitude of challenges. The identification of targets for the Ca^2+^ signals in the physiological and the pathological context remains a compelling task.

## 4. The Role of Ca^2+^ Homeostasis Dysfunction in Microglia and Astrocytes in Alzheimer’s Disease

**Figure 2:**
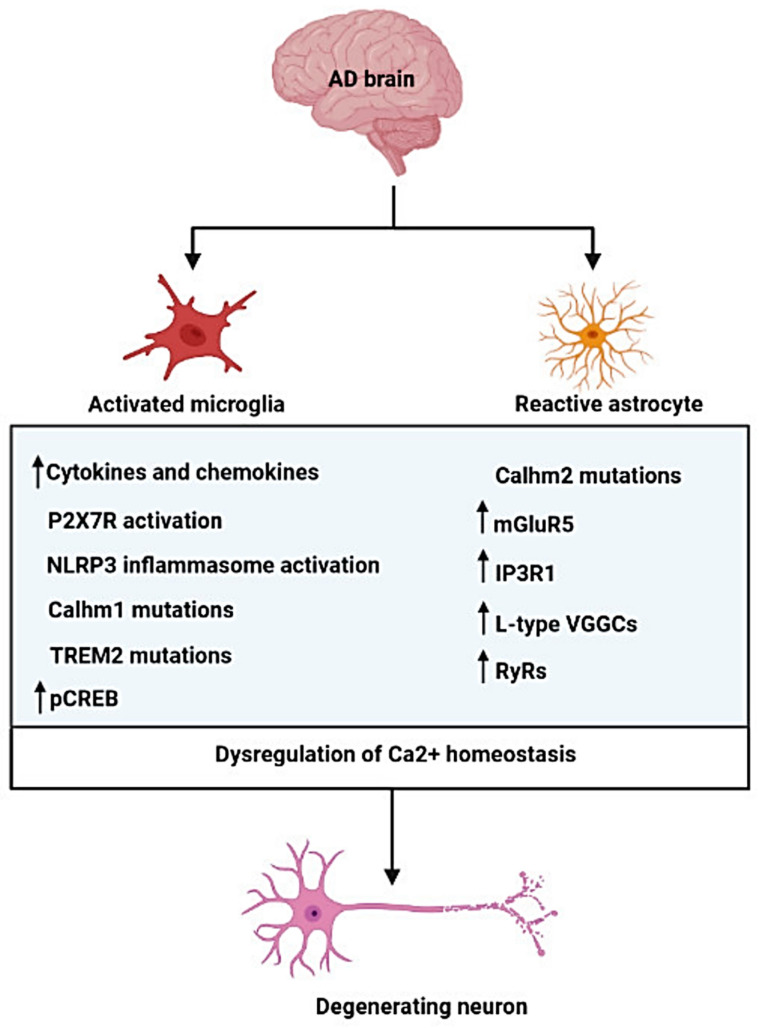
Schematic representation of Ca^2+^ homeostasis dysregulation in microglia and astrocytes in AD. P2X7R, purinergic P2X7 receptor; NLRP3, NOD-like receptor pyrin domain containing 3; Calhm1, Ca^2+^ homeostasis modulator family member 1; TREM2, triggering receptor expressed on myeloid cells 2; pCREB, phosphorylated cAMP response element binding protein; Calhm2, Ca^2+^ homeostasis modulator family member 2; mGluR5, metabotropic glutamate receptor 5; IP3R1, Inositol 1,4,5-trisphosphate (IP3) receptor type1; L-type VGGCs, L-type voltage-gated Ca^2+^ channels; RyRs, Ryanodine receptors. Created with BioRender.com; accessed on 29 July 2022.

Glial–calcium signalling acts as a molecular mechanism for integration within glial syncytium and between glial and neuronal circuits [117]. Quite naturally, this powerful signalling system also plays an important role in neuropathology, such as AD [68,69] (Figure 2). The highly dynamic intra- and intercellular Ca^2+^ signalling of astrocytes and microglia is quickly perturbed under neuropathological conditions, determining the change in cellular response and transformation into reactive phenotypes [118,119,120].

### 4.1. Microglial Ca^2+^ Signaling in Alzheimer’s Disease

Microglial cells from AD patients exhibit significant abnormalities in Ca^2+^-mediated signals compared with non-demented brains [19,121]. In fact, it has been reported that stimulation of microglia cultured with Aβ25–35 peptide results in a transient increase in intracellular Ca^2+^ [122,123]. Many microglia functions are mediated by Ca^2+^ ions [124,125,126]. In particular, microglia reaction is accompanied with intracellular Ca^2+^ increase, a process that is required to induce the release of cytokines and chemokines [127]. McLarnon et al. [19], have showed significant perturbations in Ca^2+^-mediated signal transduction (i.e., global elevation of basal levels of Ca^2+^, rapid depletion of Ca^2+^ from endoplasmic reticulum, diminished response to ATP stimulation) in microglia from AD patients.

Moreover, an increased Ca^2+^ response to a purinergic P2X7 receptor (P2X7R) agonist was found in cultured foetal human microglial cells exposed to Aβ1-42 and, consistently, enhanced P2X7R expression was stated in AD microglia [128,129]. Aβ-dependent mitochondrial toxicity in mouse microglia requires P2X7R expression and is prevented by nimodipine, a potent L-type calcium channel (LTCC) inhibitor [130].

Aberrant activation of the NLRP3 inflammasome was observed in AD; therefore, fine tuning of its activity appears essential for maintenance of proper cellular homeostasis [131,132,133,134]. NLRP3 knockout, or a dysfunction of its key signalling components significantly reduces Aβ-induced microglial activation in vitro [135], decreases Aβ deposition or Tau pathology, and also alleviates cognitive impairment in AD mouse models [136,137]. Aβ increases [Ca^2+^]i levels, which, in turn, contribute to the activation of the NLRP3 inflammasome in microglia [130]. Lee et al. [138] reported that the Ca^2+^-sensing receptor (CaSR) activates the NLRP3 inflammasome by increasing [Ca^2+^]i levels and decreasing cyclic adenosine 3′,5′-monophosphate (cAMP) levels.

Recently, the role of Ca^2+^ homeostasis modulator family proteins (Calhm, Calhm1, Calhm2, and Calhm3) has gained increasing attention in the field of AD research [139]. Calhm1 controls Ca^2+^ homeostasis, Aβ production, and neuronal cell vulnerability to Aβ-induced toxicity. Moreover, the P86L mutation of Calhm1 is correlated with the incidence of AD [140]. Calhm2, which is highly expressed in the murine brain, could regulate ATP release in astrocytes and its deletion induces a depression-like phenotype in mice [141]. In a recent study, it was shown that the expression of Calhm2 is increased in microglia of an AD mouse model [142]. In particular, in 5×FAD mice carrying five familial AD gene mutations, both conventional knockout of Calhm2 and conditional microglial knockout of Calhm2 significantly reduce Aβ deposition, neuroinflammation, and cognitive impairments. Knockout of Calhm2 inhibited microglial proinflammatory activity, but increases phagocytic activity, leading to restoration of the balance between inflammation and phagocytosis. Moreover, knockout of Calhm2 reduces acute LPS-induced neuroinflammation [142].

In addition, changes in properties of store-operated Ca^2+^ entry in microglia could lead to altered immune cell response and neurovascular unit dysfunction in the inflamed AD brain [143]. The membrane protein TREM2 regulates key microglial functions including phagocytosis and chemotaxis. Loss of function variants of TREM2 are associated with increased risk of AD [144]. Loss of TREM2 confers a defect in microglial Ca^2+^ response to purinergic signals, suggesting a window of Ca^2+^ signalling for optimal microglial motility [145]. An increase in microglial [Ca^2+^]i is linked to phosphorylation of cAMP response element-binding protein (CREB) and the activation of the pCREB pathway. In turn, CREB phosphorylation leads, for example, to the microglial secretion of proinflammatory cytokines, such as IL-6, and other molecules such as (Brain-derived neurotrophic factor) BDNF [125].

### 4.2. Astrocytic Dysregulation of Ca^2+^ Homeostasis in Alzheimer’s Disease

Recent studies have reported the existence of a causal relationship between astrocytes and neuroinflammation [10]. Alteration of Ca^2+^ homeostasis in astrocytes has been regarded as an important component of AD [146]. Aβ disrupt Ca^2+^ homeostasis, specifically in hippocampal astrocytes by targeting key elements of Ca^2+^ signalling, such as mGluR5 and IP3R1 [147]. Consistently, several studies reported that Aβ triggered transient [Ca^2+^]i increases or oscillations in astrocytes [148,149,150].

Takano et al. [151] reported that after the injection of Aβ in both Dutch/Iowa mice and control mice, the frequency of astrocytic Ca^2+^ oscillations increased significantly and became approximately three times more than the control group.

Generally, Ca^2+^ entry into astrocytes includes active transport by different types of voltage gated calcium channels (VGCCs) distributed in the membrane and passive leakage [152]. Upregulation of L-type VGCCs expression in astrocytes has been shown in pathological conditions, such as those related to AD [153]. Anekonda et al. [154] have demonstrated that L-type Ca^2+^ current blockers can protect cells from the inductive effect of Aβ. Ahmadpour et al. [152] confirmed the efficacy of blocking VGCCs, but the recovery was not significant enough.

Aβ has been shown to impair the membrane permeability, due to the Aβ-formed pores resulting in an increase in Ca^2+^ influx through the membrane [155]. This additional influx leads to damage to astrocytic homeostasis in simulations. Especially, when oscillations disappear and the astrocyte Ca^2+^ level is at a high steady state, Aβ can cause dramatic increases in [Ca^2+^]_i_. In fact, channel formation has been already proposed as a molecular mechanism for Aβ toxicity in the early 1990s [156].

Although disruptions on the membrane by Aβ are believed to be an important mechanism, the intracellular signalling pathways also deserve attention. In particular, the toxic effects of Aβ increase the channel opening probability of RyRs [157]. In fact, several studies also suggest that Aβ can directly increase the RyR expression [158,159]. In addition, Oulès et al. [160] have reported that RyR-mediated Ca^2+^ release can be reduced after treatment with RyR inhibitor.

### 4.3. How Does Aβ Disrupt Ca^2+^ Homeostasis?

Since it is well known that Aβ can disrupt RyR-regulated Ca^2+^ signals, the mechanisms for how this happens remains controversial [161,162,163]. In fact, the influence of Aβ on individual pumps, channels, and exchangers remains largely unknown [164,165]. Although it is unclear how Aβ disrupts intracellular Ca^2+^ homeostasis, there is growing evidence that Aβ directly affects the production of IP3 [166,167], calcium-induced calcium release through RyR [168,169], and the induction of Ca^2+^-permeability across membrane pores [170,171].

Notably, it was reported that the accumulation of Aβ oligomers can trigger aberrant intracellular Ca^2+^ levels by disrupting the intrinsic Ca^2+^ regulatory mechanism within cells [172,173]. These disruptions can cause changes in homeostasis levels that can have detrimental effects on cell function and survival. Although studies have shown that Aβ can interfere with Ca^2+^ fluxes, the complexity of these interactions remains elusive [174,175]. The formation of Aβ plaques and fibrils are a consequence of the imbalance between the formation and sequestration of Aβ [176,177]. Consequently, the slow accumulation of Aβ peptides can alter Ca^2+^ signalling processes within neurons and glial cells, leading to synaptic failure and neuronal death [178,179,180]. Moreover, the accumulation of Aβ has been linked to the progression of AD by altering Ca^2+^ signalling processes, but the underlying mechanisms for how and why this occurs are not fully understood [167,181].

The above-reported data indicate that Ca^2+^ dysregulation in AD occurs as a result of hyperactivity of Ca^2+^ channels in the plasma membrane and in intracellular compartments. Moreover, it seems to be restricted not only to neurons, but rather it represents a global phenomenon that affects many cell types in the brain. These observations suggest that the identification of molecules which affect Ca^2+^ homeostasis dysfunction in microglia and astrocytes could be a promising new therapeutic target in the treatment of AD.

## 5. Conclusions

Persistent and self-fuelling inflammatory processes represent the major driving force that characterize the progressive nature of AD. Microglia and astrocytes represent critical actors of neuroinflammatory response, as their interaction play a crucial role at different stages of brain pathology.

Sentinel microglia are highly active surveyors of their microenvironment, and upon detecting pathogens or damage, become activated via the common molecule signals (i.e., TNF-α, IL-1α, and C1α) to trigger reactive astrocytes. Astrocytes amplify neuroinflammation, due to their function and strategic position to mobilize peripheral immunity.

The aberrant glial cell biology seen in AD might be involved in the disruption of Ca^2+^ homeostasis in neurons, which negatively impacts on functioning and viability of neuronal cells, leading to negative functional outcomes and cognitive decline. As glia cell-mediated inflammatory responses and Ca^2+^ dysregulation in AD share a common language, it appears reasonable to hypothesize a crucial role of the latter in the neurodegenerative processes associated with dementia.

In such scenario, targeting Ca^2+^-glia-AD axis may result beneficial for AD therapy in terms of delayed disease progression but, given the limited amount of evidence currently available, further investigations are needed to determine the paths of all actors in this promising field of research.

## Data Availability

Not applicable.
